# High wall shear stress-dependent podosome formation in a novel murine model of intracranial aneurysm

**DOI:** 10.3389/fstro.2024.1494559

**Published:** 2024-11-25

**Authors:** Jiayi Lu, Mengjun Dai, Yuanqing Yan, Louise D. McCullough, Yan-Ning Rui, Zhen Xu

**Affiliations:** 1 Department of Neurosurgery, McGovern Medical School, The University of Texas Health Science Center at Houston, Houston, TX, United States; 2 Department of Surgery, Northwestern University, Chicago, IL, United States; 3 Department of Neurology, McGovern Medical School, The University of Texas Health Science Center at Houston, Houston, TX, United States

**Keywords:** murine model, cerebrovascular integrity, intracranial aneurysm, high wall shear stress, endothelial dysfunction, podosomes

## Abstract

High wall shear stress (HWSS) contributes to intracranial aneurysm (IA) development. However, the underlying molecular mechanisms remain unclear, in part due to the lack of robust animal models that develop IAs in a HWSS-dependent manner. The current study established a new experimental IA model in mice that was utilized to determine HWSS-triggered downstream mechanisms. By a strategic combination of HWSS and low dose elastase, IAs were induced with a high penetrance in hypertensive mice. In contrast, no IAs were observed in control groups where HWSS was absent, suggesting that our new IA model is HWSS-dependent. IA outcomes were assessed by neuroscores that correlate with IA rupture events. Pathological analyses confirmed these experimental IAs resemble those found in humans. Interestingly, HWSS alone promotes the turnover of collagen IV, a major basement membrane component underneath the endothelium, and the formation of endothelial podosomes, subcellular organelles that are known to degrade extracellular matrix proteins. These induced podosomes are functional as they degrade collagen-based substrates locally in the endothelium. These data suggest that this new murine model develops IAs in a HWSS-dependent manner and highlights the contribution of endothelial cells to the early phase of IA. With this model, podosome formation and function was identified as a novel endothelial phenotype triggered by HWSS, which provides new insight into IA pathogenesis.

## Introduction

An intracranial aneurysm (IA) is a localized dilation of blood vessels that frequently occurs at the circle of Willis (CoW), the arterial network at the base of the brain. The rupture of IA causes subarachnoid hemorrhage that is responsible for over 70% of mortality and morbidity in these patients (Xu et al., 2019). Unfortunately, there are no drug treatments for IAs except for two surgical interventions such as aneurysm clipping or endovascular coiling. The identification of novel therapeutic targets is greatly impeded by the limited knowledge regarding the molecular and pathologic mechanisms that contribute to IA development.

Over the past decades, animal models have been developed in different species in order to understand the underlying molecular mechanisms of IA pathogenesis. IAs were successfully induced in mice by applying high wall shear stress (HWSS), a frictional force directly acting on the luminal side of vessel walls and a known risk factor for IA. However, the downstream molecular events triggered by HWSS remain largely unknown due to several limitations of the extant models. In earlier studies HWSS leads to IA formation in hypertensive mice (Morimoto et al., 2002). However, the induced IAs were small and mainly identified at the same location of the CoW, suggesting that this model may recapitulate a subset of human IAs. Although large IAs were found in another mouse model (Hosaka et al., 2014), the specific contribution of HWSS to IA development is unknown. Such a knowledge gap can be filled by the development of a new murine model that forms large IAs sharing a broad spectrum of pathology as seen in humans as well as in a HWSS-dependent manner.

Here, we developed a novel mouse model by combining HWSS with two additional factors that facilitate IA development. Within 2–3 weeks, large IAs were detected at different sites of CoW with a high penetrance. Histological analyses confirmed that these experimental IAs resemble those in humans. Importantly, IAs were not detected in all control groups in the absence of HWSS, demonstrating that HWSS plays an essential role in IA development. Using this model, we explored HWSS-induced vascular changes that contribute to IA development at the early stage. Manipulating these early events could provide novel therapeutic targets for IA disease.

## Methods

### Mouse model of intracranial aneurysm

All animal care procedures and experimental protocols were approved by the Institutional Animal Care and Use Committee and were performed in agreement with the National Research Council Guide for the Care and Use of Laboratory Animals. A certified animal care technician in the Center for Laboratory Animal Medicine and Care (CLAMC) at UTHealth performed daily checks and weekly thorough examinations on the wellness of animals and provided recommendations on any animals showing signs of illness or distress. Any mice that became distressed and moribund were euthanized prior to sustained morbidity via institutionally approved house line CO_2_-mediated asphyxiation, followed by cervical dislocation as a secondary method.

C57BL/6J mice (8- to 10-week-old, Jackson Laboratory) were used in the study. Mice were randomized into sham or LCCA ligation. The same individual performed all surgeries, and investigators responsible for IA assessment, functional assays and data analyses were blinded to experimental groups. Sample sizes were chosen empirically in the absence of prior experience with carotid artery ligation technique in our lab.

IAs were induced by a combination of left common carotid artery (LCCA) ligation and elastase injection in hypertensive mice. All surgeries were performed in mice anesthetized by isoflurane [4% induction, 1.5% maintenance, in medical air enriched with oxygen (25%)]. For LCCA ligation, the skin along the ventral side of the neck was shaved and a midline incision was made. The blunt dissection of the distal LCCA was performed and LCCA was tied off just proximal of the carotid bifurcation with the use of 6–0 suture. Sham operation was performed as a surgical control with LCCA isolated but not ligated. Elastase (E7885, Sigma Aldrich) was injected stereotaxically into the right basal cistern as described in a previous publication (Nuki et al., 2009). In brief, anesthetized mice were mounted in a rodent stereotaxic frame (model 940 small animal stereotaxic instrument with a digital display console, David Kopf Instruments, Supplementary Figure 2B) (Nuki et al., 2009; Lu et al., 2019, 2021). The skin was opened to uncover the skull and expose bregma, lambda, and the location of the desired injection site. Small drill holes were made in the skull. The microinjector was loaded with 2.5 μl of the elastase solution (12 milli-units dissolved in PBS) and then lowered at the right basal cistern (AP: −2.5 mm, ML: +1 mm, DV: −5.0 mm). The elastase solution was infused at a rate of 0.2 μl/min. To induce hypertension, an osmotic pump (Model 1002, Alzet pump, Durect Corp.) containing angiotensin-II (A9525, Sigma Aldrich, 750 ng/kg/min dissolved in PBS) was implanted under the dorsal skin.

Mice were fixed by intracardial perfusion of 4% paraformaldehyde and IAs were visualized by a continuous perfusion of a solution containing both bromophenol blue dye (2 mg/ml in PBS) and gelatin (20% in PBS). IAs were defined as localized outward bulging of the vascular wall with the largest diameter greater than that of the parent arteries. Pictures of the major cerebral arteries were taken by Leica S9 stereo microscope. The brain tissues were further fixed with 4% paraformaldehyde for 24 h and then dehydrated in 30% sucrose overnight at 4°C for cryosection (Rui et al., 2015b).

### Intracranial aneurysm outcomes

Neurological score and body weight were monitored daily. Neurological symptoms were scored: 0, normal function; 1, reduced activity or weight loss >2 g of body weight (≈10% weight loss) for 24 h; 2, flexion of the torso and forelimbs upon lifting the animal by the tail; 3, circling to one side with normal posture at rest; 4, leaning to one side at rest; 5, no spontaneous activity; and 6, sudden spontaneous death.

### Blood flow measurement by ultrasound scanner

Blood flow in LCCA (ligated side) and the right CCA (flow-augmented side) was measured 5 mm below the carotid bifurcation with an ultrasonic instrument (Vevo 3100 Preclinical Imaging System, FUJIFILM VisualSonics). Each mouse was anesthetized under isoflurane anesthesia (2%) and monitored to maintain heart rate above 500 beats/min during measurements. Blood flow was obtained using the pulse wave Doppler.

### Histological and immunofluorescence analyses

Each whole brain was sectioned serially into 10-um coronal sections using a cryostat (Leica CM1860). The sections were treated with different staining methods including hematoxylin and eosin (H&E), Verhoeff-Van Gieson (Cat#: 25089–1, Polysciences), and Masson’s Trichrome (Cat#: NC9900705, StatLab). The following antibodies were used for immunofluorescent analyses on vascular cells: anti-mouse CD45 (MABF1466, EMD Millipore) for pan-inflammatory cells, anti-mouse F4/80 (14–4801-81, Invitrogen) for macrophages, anti-mouse CD31 (557355, BD Pharmingen) for endothelial cells, and anti-α-smooth muscle actin (14–9760-82, Invitrogen) for smooth muscle cells. Alexa 488 or 594-conjugated secondary antibodies were utilized and fluorescent images were taken under Leica TCS SP5 confocal microscope.

### *En face* immunostaining

To detect podosome formation in intimal endothelial cells of the circle of Willis, *en face* immunostaining was conducted by combining two protocols (Ko et al., 2017; Hur et al., 2016). Two or three days after LCCA ligation or sham operation, the mouse circle of Willis was dissected out under the stereo microscope using mini-Vanna scissors as described in previous publication (Hur et al., 2016). The entire circle of Willis was incubated in a 1:200 dilution of primary antibody against podosome markers such as cortactin (05–180-I-100UL, EMD Millipore), p-SRC-Y416 (44–660G, ThermoFisher Scientific), and endothelial marker VE-Cadherin (555289, BD Biosciences) at 4°C overnight. Alexa 488 or 594-conjugated secondary antibodies were utilized and *en face* preparations of each segment of the circle of Willis were performed as described in a previous publication (Ko et al., 2017). Fluorescent images were taken under a Leica confocal microscope and further quantified by ImageJ as outlined in our previous publications (Rui et al., 2015a,b).

### *In situ* zymography

To detect gelatinase activity, specifically MMP activity after LCCA ligation, we conducted *in situ* zymography using gelatin as a substrate according to the previous study (George and Johnson, 2010; Galis et al., 1995; Mook et al., 2003). Briefly, non-fixed brain tissue section was incubated in the presence of fluorescent dye-quenched gelatin/DQ-gelatin (D12054, ThermoFisher Scientific) in 1X zymogram development buffer (LC2671, ThermoFisher Scientific) at 37°C for 12 h in a dark humid chamber. Upon gelatinase cleavage, fluorescence was released that directly reflected the level of the gelatinase activity by the tissue. Fluorescent images were obtained using Leica confocal microscope.

### Western blotting

Mouse tissues such as the circle of Willis were dissected under a Leica S9 microscope followed by a quick wash in 1X PBS. The circle of Willis segments was dissolved in a Triton X-100 lysis buffer as described in a previous publication (David-Morrison et al., 2016; Rui et al., 2004) and sonicated briefly before centrifugation at 13,000 rpm for 30 min at 4°C. Triton-soluble cell lysates were added by 2X SDS sample buffer. The Triton-insoluble pellets were further treated by pepsin (P7012, Sigma Aldrich, 1 mg/ml in 0.5 M acetic acid) for 48 h at 4°C with gentle swirling as in a previous publication (Boudko et al., 2018). The re-dissolved basement membrane matrix proteins in the supernatants were denatured in the SDS sample buffer. Both Triton-soluble and -insoluble fractions were subjected to SDS–PAGE electrophoresis and transferred to nitrocellulose membranes from Millipore. After blocking with 5% non-fat milk in Tris-buffered saline with 0.1% Tween-20 for 1 h, membranes were incubated with primary antibodies against Collagen I (14695–1-AP, ThermoFisher Scientific), Collagen IV (2150–1470, Bio-Rad), or GAPDH (sc-32233, Santa Cruz Technology). Secondary antibodies conjugated with Alexa-800 or Alexa-680 (Invitrogen) were used. The signals were detected by the Odyssey Infrared Imaging System and quantified by Image Studio Lite associated with the Odyssey system as described in previous publications (David-Morrison et al., 2016; Rui et al., 2015a,b).

### Quantitative RT-PCR

Mouse tissues such as the circle of Willis were dissected under a Leica S9 microscope followed by a quick wash in 1X PBS. The circle of Willis segments were dissolved in TRIzol and total RNA were isolated using TRIzol^™^ Plus RNA Purification Kit (12183555, ThermoFisher Scientific). Quantitative RT-PCR were performed based on the SYBR green protocol as described in a previous publication (Rui et al., 2020). The primers used are listed as follows:

MMP1: 5′- AGGAAGGCGATATTGTGCTCTCC/5′- TGGCTGGAAAGTGTGAGCAAGC

MMP2: 5′- CAAGGATGGACTCCTGGCACAT/5′- TACTCGCCATCAGCGTTCCCAT

MMP14: 5′- GGATGGACACAGAGAACTTCGTG /5′- CGAGAGGTAGTTCTGGGTTGAG

ACTA2: 5′- TGCTGACAGAGGCACCACTGAA /5′- CAGTTGTACGTCCAGAGGCATAG

MYH11: 5′- GCAACTACAGGCTGAGAGGAAG /5′- TCAGCCGTGACCTTCTCTAGCT

TAGLN: 5′- GCAGATGGAACAGGTGGCTCAA /5′- CCCAAAGCCATTAGAGTCCTCTG.

### RNA-seq profiling

Total RNA was extracted from human brain microvascular endothelial cells (HBMECs) in a 100 mm cell culture dish by TRIzol as described in a previous publication (Rui et al., 2020). The poly-A containing mRNA molecules were concentrated using poly-T oligo-attached magnetic beads. Quantitative RNA-seq profiling was performed by a next-generation sequencing (NGS) core facility in UT medical branch (Galveston). The abundance of individual genes was indicated by the value of Fragments Per Kilobase Million (FPKM).

### Statistical analysis

All *in vitro* experiments were performed in triplicates and repeated at least three times. All assays were performed by an investigator blinded to the treatment group. The statistical significance of the difference between experimental groups was determined by two-tailed unpaired Student’s *t*-test, or one-way analysis of variance followed by Tukey’s *post hoc* test. Survival was analyzed with log rank (Mantel-Cox) test. Differences were considered significant for *p* < 0.05 (noted in the figures as *). Data are presented as mean + SEM from a minimum of three independent experiments. The sample size (*n*) is indicated in each figure and corresponds to individual experiments unless otherwise stated. Statistical analysis was performed by Prism 9 (GraphPad Software, San Diego, CA).

## Results

### High wall shear stress (HWSS) is essential for IA induction

**FIGURE 1:**
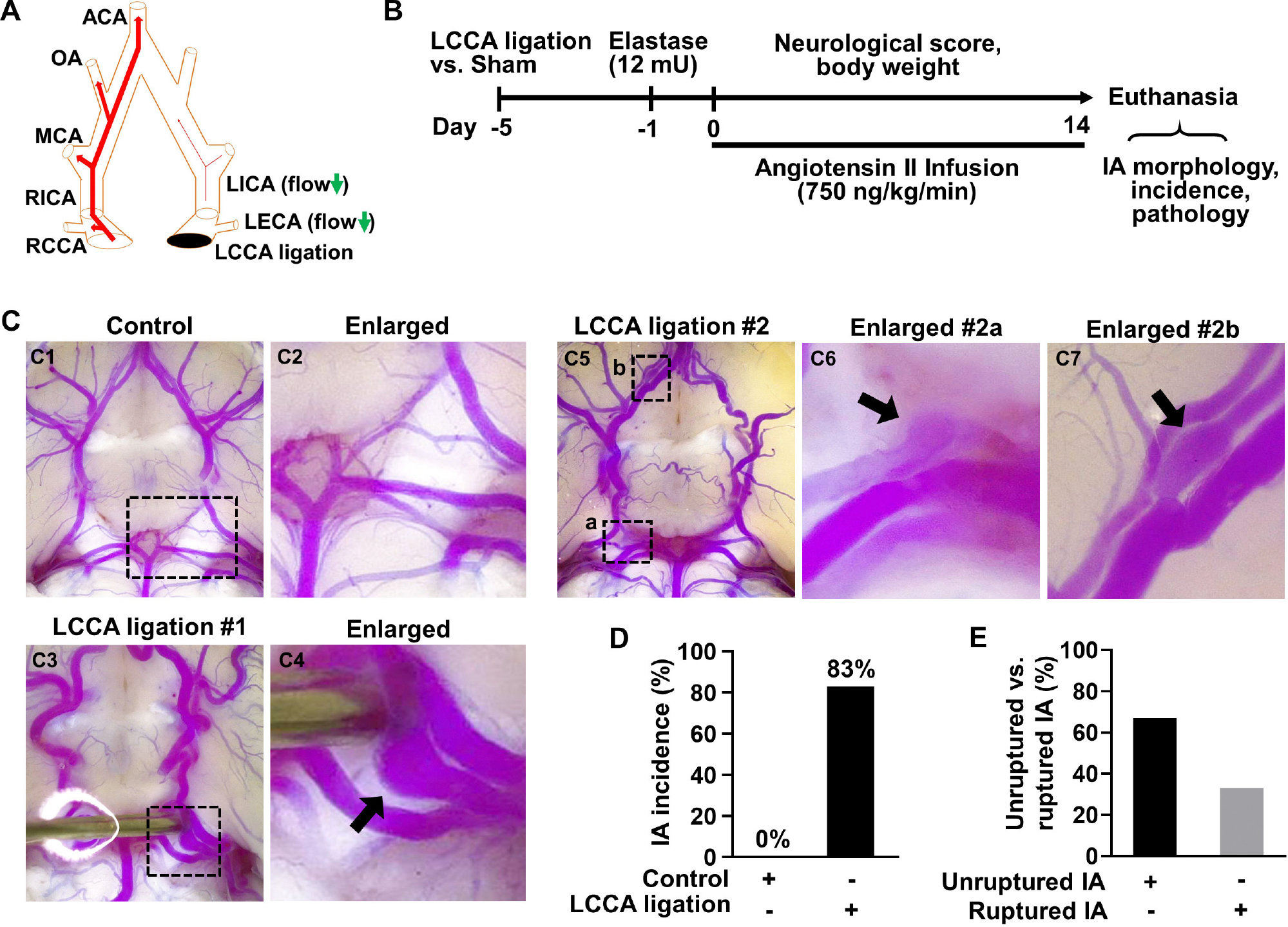
HWSS is a major trigger for IA formation in mice. **(A)** A schematic view of the left common carotid artery (LCCA) ligation model. CCA, common carotid artery; ECA, external carotid artery; ICA, internal carotid artery; MCA, middle cerebral artery; OA, olfactory artery; ACA, anterior cerebral artery. **(B)** An experimental protocol for IA induction and assessment in mice. All mice underwent LCCA ligation or sham operation followed by a stereotaxic injection of elastase (12 mU) into the right cistern magna. After infusion of angiotensin-II (750 ng/kg/min), daily neurological scores and body weight were recorded and IA-related assays were performed after all mice were euthanized due to severe neurological deficits or at the end of two weeks. **(C)** LCCA ligation (IA group) vs. sham operation (control group) were performed in mice followed by elastase injection and angiotensin infusion indicated in **(B)**. After IA induction with sham operation (*n* = 8) or LCCA ligation (*n* = 10), cerebral arteries in the circle of Willis (CoW) were visualized by vascular perfusion of bromophenol blue/gelatin mixture. A representative image of cerebral blood vessels from control group was shown in **(C1, C2)**. Representative images were shown of one IA at the left posterior communicating artery **(C3, C4)** or of multiple IAs **(C5–C7)** at different locations from LCCA-ligated group. **(D)** The rate of IA incidence in mice with sham operation or LCCA ligation. Four mice were excluded as their brains were too soft to be help up for postmortem analyses after sudden death. **(E)** The comparison between unruptured and ruptured IA formation rate in LCCA-ligated mice.

It is well documented that HWSS can be created by unilateral ligation of the common carotid artery (Gao et al., 2008; Morimoto et al., 2002). After the ligation of left common carotid artery (LCCA), the blood flow in the contralateral arteries in the circle of Willis (CoW) increases due to the compensatory effects (Figure 1A and Supplementary Figure 1). Such a method has been used to induce IAs in mice. However, the induced IAs are small (electronic microscopy is required for further characterization), mainly locate at the same bifurcation, and require about 4 months to develop with no rupture events reported (Morimoto et al., 2002). Therefore, this model may reflect a subset of human IAs.

To overcome these limitations, we developed a new method to induce IAs in mice in a HWSS-dependent manner (Figure 1B). HWSS was introduced by LCCA ligation, which was performed under a dissection microscope (Supplementary Figure 2A) and confirmed by a dramatic reduction of blood flow velocity based on the high-frequency ultrasound imaging (Supplementary Figures 3A, B). To facilitate IA progression in the CoW, a low dose of elastase (12 mU) was injected stereotaxically into the cerebral spinal fluid at right basal cisterna (Supplementary Figure 2B). Elastase is known to degrade elastin, a major component in internal elastic lamina (IEL) that separates intima from media. The loss of IEL is a hallmark trait for all IAs in humans. Indeed, we observed a certain degree of dolichoectasia and tortuosity in the CoW from the groups treated with elastase (Supplementary Figures 4A2, A4, A6), indicating that IEL was partially degraded. In addition, hypertension was created by angiotensin infusion, a widely used method to increase blood pressure in various animals. Following the completion of IA induction-related surgeries, we evaluated its outcomes including overall survival, neurological symptoms, and subarachnoid hemorrhage. In addition, we examined IA morphology and incidence as end-point assays (Figure 1B).

To visualize IAs, we perfused the mice intracardially with bromophenol blue dye in pre-warmed gelatin solution as previous reported (Nuki et al., 2009). A saccular IA was observed in the posterior communicating artery (Figures 1C3, C4), while no IAs were detected in the CoW of control mice (Figures 1C1, C2). We also observed more than one IAs in LCCA-ligated mice such as those in the right posterior communicating artery and olfactory artery, respectively (Figures 1C5–C7). Of note, the aneurysm in Figure 1C6 was partially thrombosed as the blue dye/gelatin mixture cannot fill up the entire aneurysmal sac. These observations are consistent with IA multiplicity and thrombosis often seen in patients (Jabbarli et al., 2018; Ngoepe et al., 2018). Overall, IAs developed in 83% of mice receiving LCCA ligation while none were detected in the control group (Figure 1D), suggesting that HWSS is essential for inducing IAs in our mouse model. To corroborate this conclusion, we examined IA formation in all the other control groups that underwent either single or double surgeries (Supplementary Figure 4A). IAs were not found in them (Supplementary Figure 4B), further supporting that HWSS is a driving force for IA development in our model.

**FIGURE 2:**
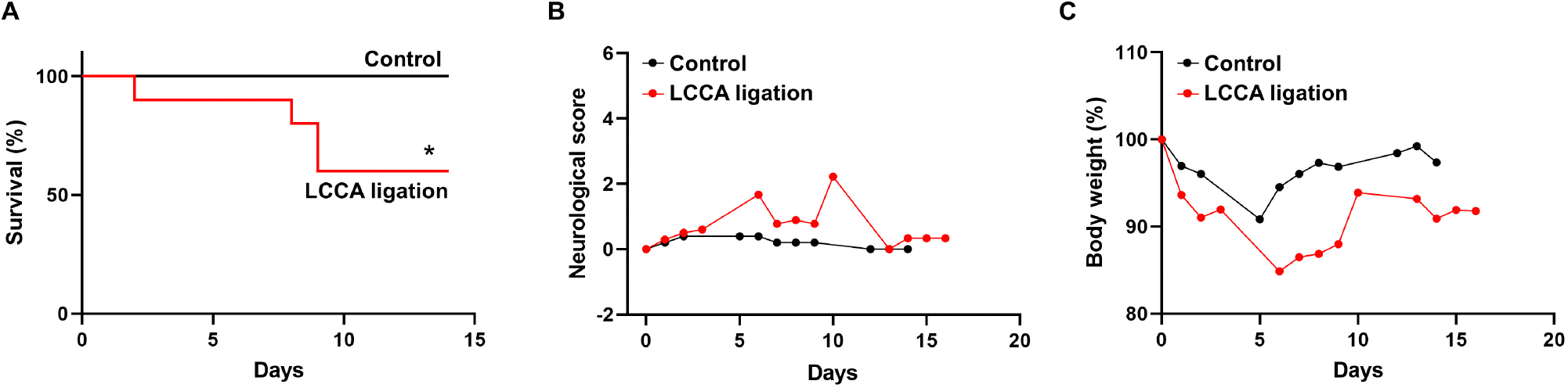
Experimental IA outcomes. **(A–C)** Mouse survival curve, neurological deficit scores, and body weight were recorded as indicated in both sham-operated (*n* = 8, black line or curve, control group) and LCCA-ligated mice (*n* = 10, red line or curve, IA group) after the completion of IA induction surgeries (*p < 0.05).

In humans, unruptured IAs are generally asymptomatic and SAHs due to ruptured IAs cause high morbidity and mortality. As such, we evaluated IA outcomes including neurological symptoms and survival rate during the timeline. Mice with severe neurological deficits (see methods for criteria) were euthanized then postmortem analyses were conducted to confirm SAH. Two mice were found to have massive bleeding in the subarachnoid space on day 8 and day 9 (Supplementary Figure 5). The IA rupture rate is about 33% and two-fold lower than the incidence of unruptured IA (Figure 1E), suggesting that HWSS-induced IAs in our model mimic those at different stages and are relatively stable. Consistently, the mice treated by our regimen showed a moderate death rate while all mice survived in control group (Figure 2A). We also evaluated mouse neurological behaviors daily for 2–3 weeks before euthanasia. Dichotomous outcomes were observed, i.e., neurological symptoms progressively became worse until day 6. Then mice started to recover and showed a varying degree of neurological deficits after day 9 (red curve in Figure 2B). In contrast, control mice were completely free of severe neurological deficits (black curve in Figure 2B). We reason that the mice after three surgeries need about one week to recover fully while ruptured or leaked IAs at a later time window contribute to the second phase of development of severe neurological symptoms. Consistently, mouse body weight showed a similar dichotomous distribution. Most of LCCA-ligated mice began to lose body weight, up to more than 15% (over 2 g), at day 6 and then regained it throughout the rest of the experimental timeline (red curve in Figure 2C). Since each mouse with weight loss over 2 g was scored 1, this accounts for the first phase of progressive worsening based on neuroscores, which may not be indicative of IA-related outcomes but mainly contributed by surgery-related impacts on overall physical status. In addition, the LCCA-ligated mice showed the worst outcomes compared to all other control groups (Supplementary Figures 6A–C).

### High wall shear stress-induced IAs share similar pathological features with human IAs

**FIGURE 3:**
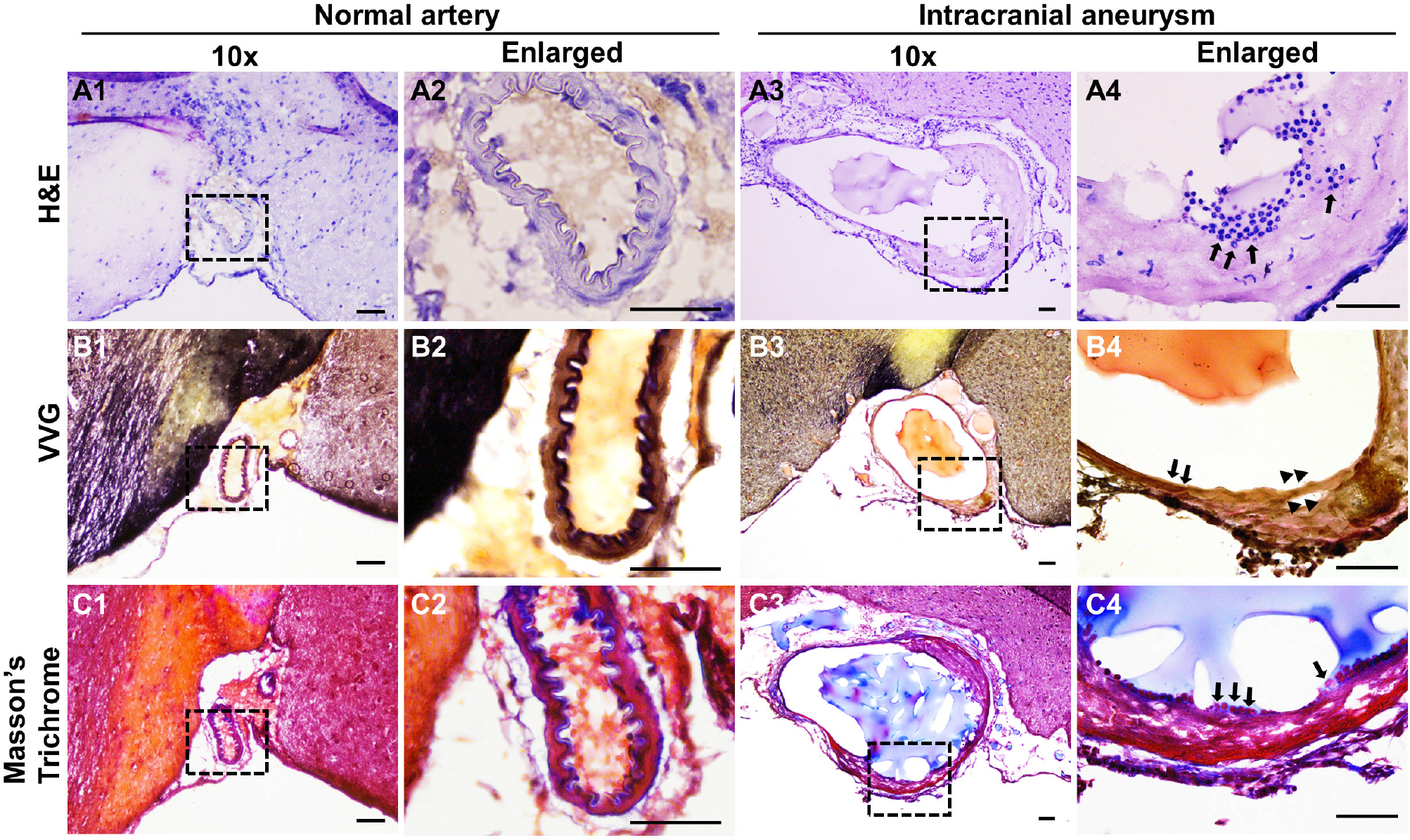
Histological analyses of the experimental IAs. **(A)** Representative light microscopic images of frozen sections with H&E (nuclei: dark blue; cytoplasm: pink) in control normal arteries **(A1, A2)** and IA samples **(A3, A4)**. **(B)** Internal elastic lamina was highlighted by Verhoeff Van-Gieson (VVG) staining (dark blue to black, **B1–B4**) in normal arteries and IA samples, respectively. **(C)** Masson-trichrome staining shows the distribution of smooth muscle cells (red in **C1–C4**). Blacked boxed regions were selected for the images in higher magnifications. *n* = 8 for each group. Scale bars: 50μm.

To examine if the induced IAs mimics those found in humans, we performed pathological analyses on the intracranial arteries from wild-type or the treated mice. Normal intracranial arteries contain an innermost endothelium separated from medial smooth muscle cells by IEL (Xu et al., 2019). The highly organized vascular structure can be viewed by hematoxylin-eosin (HE) staining (Figures 3A1, A2). However, the vascular architecture was dramatically altered in IA lesions and some segments of the vessels become thinner or thicker (Figure 3A3). A number of leukocytes accumulated in the vascular walls (black arrows in Figure 3A4). These data are consistent with clinical findings that disorganization of medial layers and infiltration of inflammatory cells frequently occur in human IA samples. Since IEL degradation is a hallmark for all IAs, we further examined IEL integrity via Verhoeff Van-Gieson staining, a method widely used to highlight the elastic tissues. In normal arteries, IEL displays as continuous wavy folds close to the intima (Figures 3B1, B2) but becomes fragmented in IA tissues (Figures 3B3, B4). We noticed that at the luminal side of discontinued IEL, there are some areas in pink and yellow, which indicate the accumulation of collagen and smooth muscle cells, respectively (arrowheads in Figure 3B4). We further utilized trichrome staining that can better distinguish collagen (blue) from smooth muscles (red). Unlike normal arteries, smooth muscle layers are disorganized with collagen randomly distributed within (Figures 3C1–C4).

**FIGURE 4:**
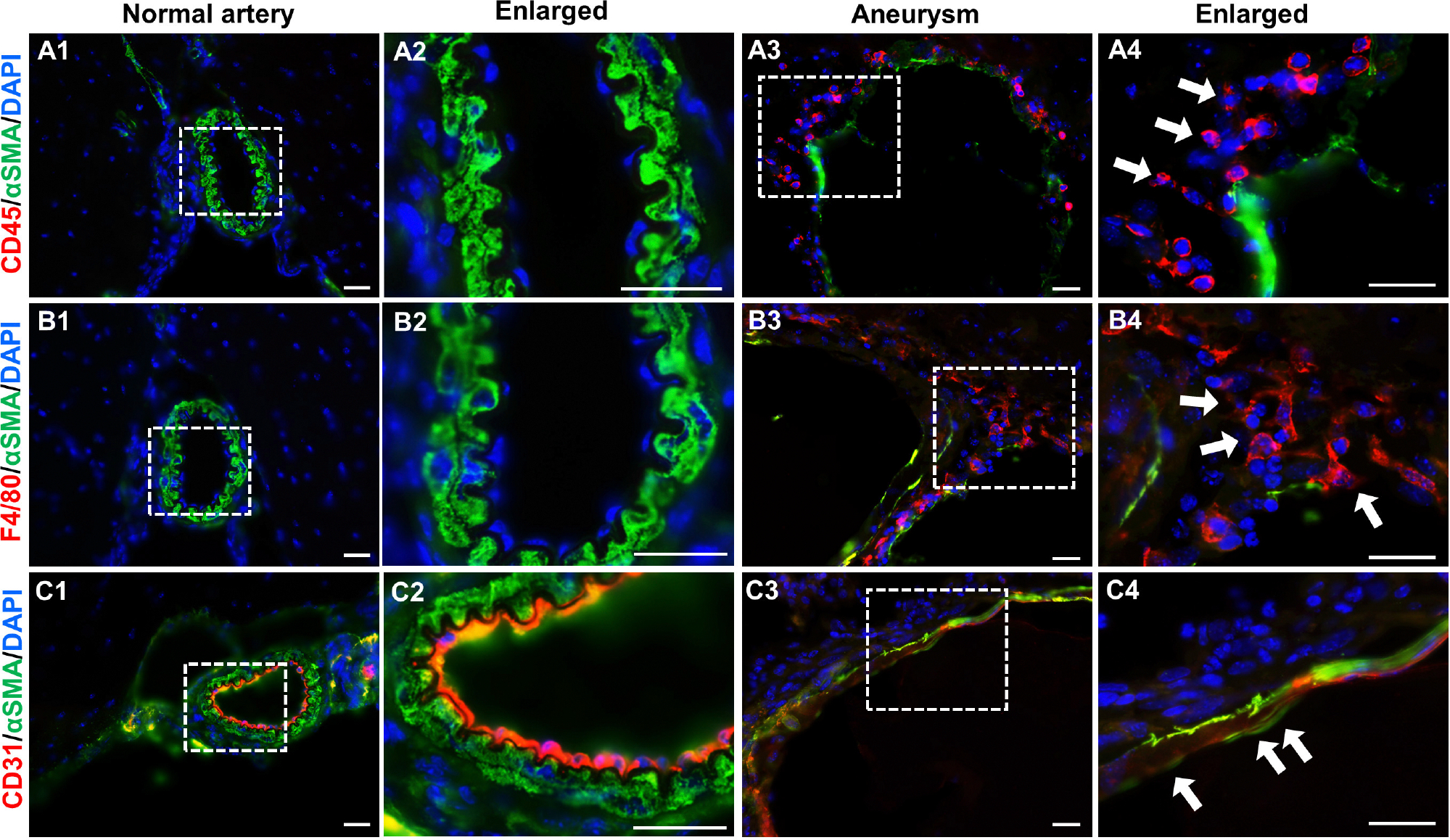
Immunofluorescence analyses of vascular cells in experimental IAs. **(A–C)** Frozen sections from control arteries or IA samples were immunostained for CD45, F4/80, CD31, and αSMA, respectively. CD45: a pan-inflammatory cell marker (red in **A1–A4**); F4/80, a marker for macrophages (red in **B1–B4**); CD31, an endothelial cell marker (red in **C1–C4**), and αSMA, a smooth muscle marker to highlight vascular structure (green in all panels). Nuclei were stained with 4′ ,6-diamidino-2-phenylindole (DAPI, blue in all panels). White boxed regions were selected for the images in higher magnifications. *n* = 5 for each group. Scale bars: 50μm.

Although histological techniques conveniently reveal overall vascular structure, their resolution is limited for defining each specific cell type. Next, we performed immunofluorescence assays using antibodies that specifically recognize markers for endothelial cells (CD31), smooth muscle cells (α-SMA), and leukocytes (CD45 for pan-leukocytes and F4/80 for macrophages). No leukocytes or macrophages were detected in normal intracranial arteries (Figures 4A1, B1). Smooth muscle cells (green) form continuous and organized layers throughout the vascular wall (Figures 4A2, B2). This data indicate that under physiological conditions vascular inflammation is low and the spindle-shaped, contractile smooth muscle cells are predominant. In contrast, IA tissues are positive for a large number of leukocytes or macrophages (red) (Figures 4A3, B3) and the smooth muscle layers in some segments are completely lost or dislocated (Figures 4A3, B3). Moreover, endothelium became discontinuous (Figure 4C4) with some areas invaded by medial smooth muscle cells (Figure 4C3) while these remained intact in controls (Figures 4C1, C2), suggesting that endothelial integrity was impaired, another hallmark of IA pathogenesis. Taken together, the IAs induced by our regimen recapitulated many features observed in human samples such as endothelial degeneration, IEL fragmentation, medial disorganization as well as localized inflammation, further validating this new model.

### High wall shear stress upregulates matrix-lytic proteins in endothelial cells

**FIGURE 5:**
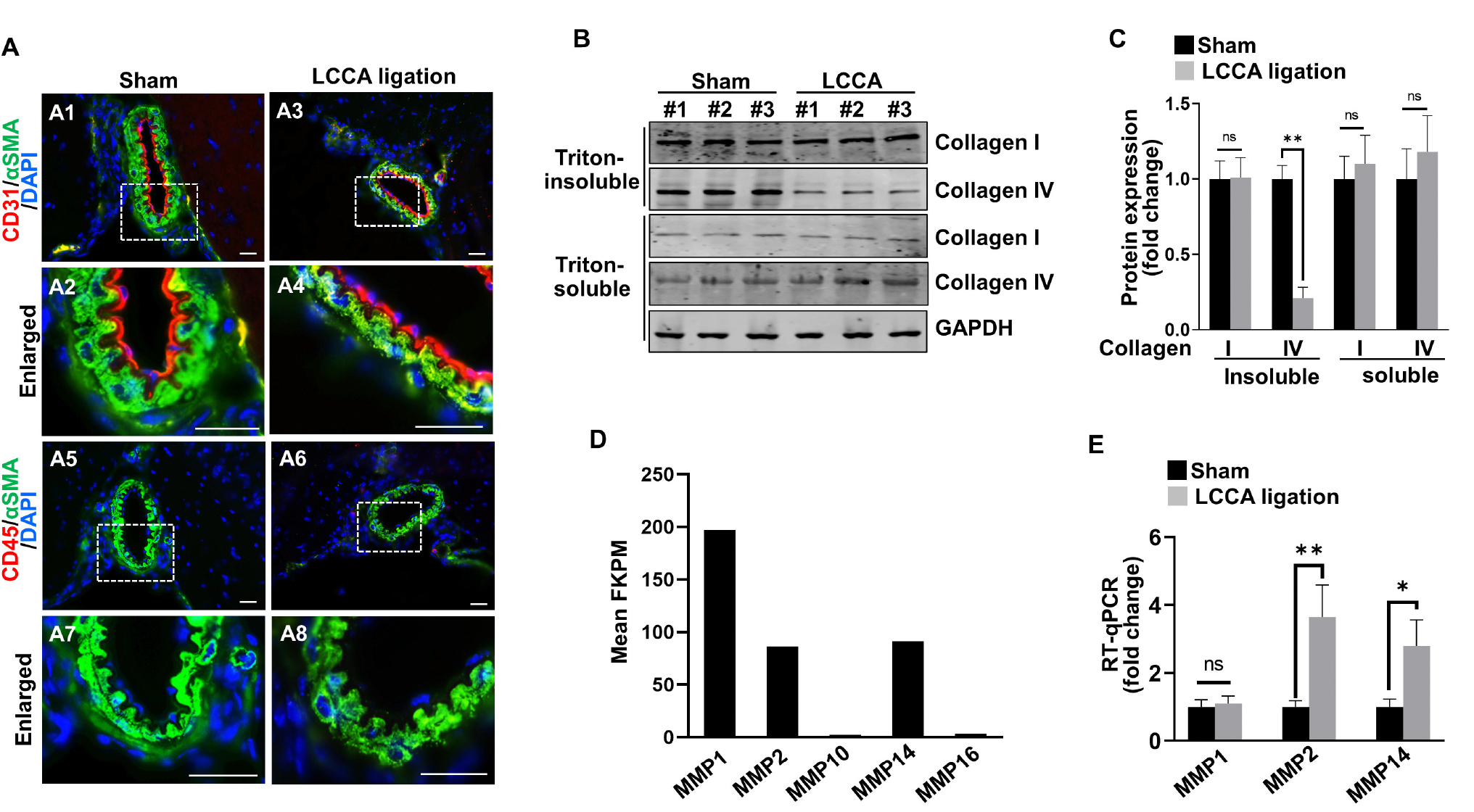
HWSS up-regulates metalloproteinases expressions and collagen IV degradation. **(A)** Immunostaining of the CoW frozen sections from sham-operated or LCCA-ligated mice (3 days after surgery) against endothelial cell marker CD31 (red) and pan-inflammation marker CD45 (red) as well as smooth muscle cell marker αSMA (green). Nuclei were highlighted by DAPI staining (blue). *n* = 6 for each group. **(B, C)** Total cell lysates (Triton soluble vs. insoluble) were extracted from the CoW in mice (3 days after surgery) under sham operation or LCCA ligation. Western blot was performed to reveal the expression of collagen I and collagen IV, respectively. The level of each protein was normalized to GAPDH loading control and further quantified in **(C)**. **(D)** Based on RNA-seq profiling, expression levels of metalloproteinases [Fragments Per Kilobase Million (FPKM) >2.0] were shown while all other metalloproteinases with FPKM lower than 2.0 were excluded. **(E)** Quantitative RT-PCR (RT-qPCR) revealed the expression levels of MMP1, MMP2, and MMP14 in the CoW that was exposed to sham operation or LCCA ligation. The expression level of each gene was normalized to GAPDH. *n* = 3 independent experiments for **(C, E)**, respectively. n.s., not significant; *p < 0.05, **p < 0.01. Scale bar: 50μm.

HWSS has been recognized as a risk factor for IA development (Shojima et al., 2004). However, the downstream events especially in the *in vivo* setting remain largely unknown. We investigated the underlying molecular mechanisms in our new model. First, we examined if HWSS induces morphological changes in intracranial vascular cells. Both intimal endothelium (red) and medial smooth muscle cell layers (green) in the CoW from LCCA-ligated mice appear similar to those from sham-operated controls (Figures 5A1–A4). No inflammation was detected (Figures 5A5–A8), consistent with previous reports that inflammatory cells are scant at the early stage of IA pathogenesis (Kolega et al., 2011).

Although vascular cells appeared normal in shape and arrangement, dysregulated intracellular function may occur prior to the morphological changes. HWSS was reported to induce the pro-matrix remodeling function in cultured endothelial cells (Dolan et al., 2012). We hypothesized that HWSS in our model negatively regulates matrix stability and thus contributes to IA development. Collagen I and IV are two major extracellular matrix proteins that surround smooth muscle cells in the media and support intimal endothelial cells in the basement membrane, respectively. Interestingly, the amount of collagen IV in Triton-insoluble fraction from the CoW was significantly reduced upon HWSS, while that in Triton-soluble fraction remained unchanged (Figures 5B, C). However, the collagen I level appears stable in either fraction after LCCA ligation. Therefore, our data suggest that there is an increased degradation of collagen IV in the basement membrane, possibly by upregulation of the matrix-lytic enzymes such as matrix metalloproteinases (MMPs). Our RNA-seq profiling demonstrated that MMP1, MMP2 and MMP14 are the three major types of metalloproteinases expressed in brain endothelial cells (Figure 5D). Furthermore, the level of MMP2 and MMP14 increased significantly upon LCCA ligation (Figure 5E), indicating that these two matrix-lytic enzymes are inducible in endothelial cells by HWSS and likely to be responsible for the degradation of collagen IV in the basement membrane. We also evaluated smooth muscle cell contraction function by measuring the expression level of different markers such as α-SMA (ACTA2), myosin heavy chain 11 (MYH11), and transgelin (TAGLN). Clearly, their expression remained stable after LCCA ligation (Supplementary Figure 7), suggesting that smooth muscle cells still predominantly display contractile phenotype upon HWSS.

### High wall shear stress induces endothelial podosome formation and function in the circle of Willis

**FIGURE 6:**
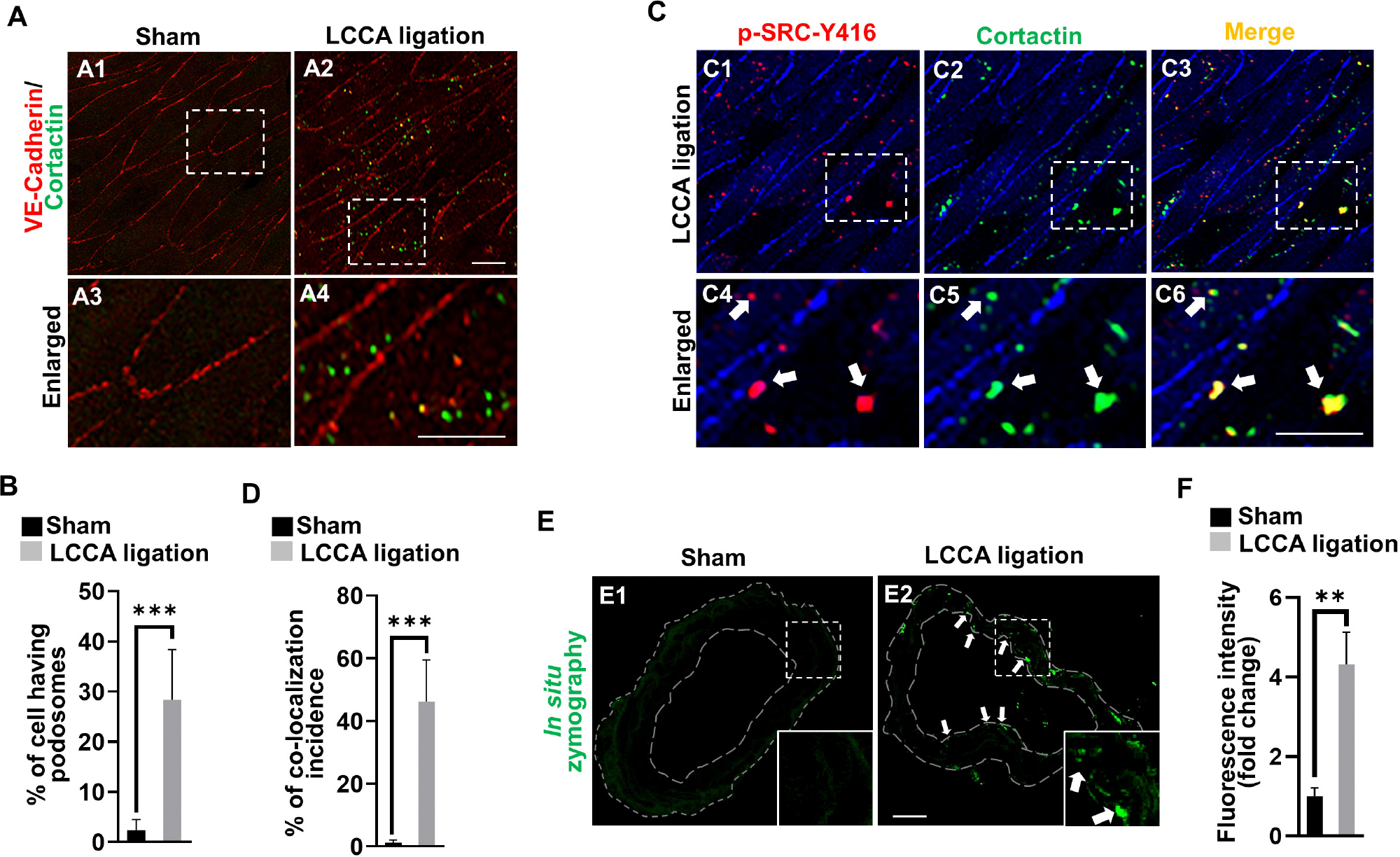
HWSS induces endothelial podosome formation and function. **(A)** Representative images of podosomes were shown by *en face* immunostaining against cortactin (green) in the dissected CoW from mice after sham operation or LCCA ligation. Endothelial lining was labeled by anti-VE-cadherin antibody (red). **(B)** The rate of podosome formation was calculated by the number of cells having podosomes over the total number of cells counted. At least nine different fields under 20X objective were selected for calculations. *n* = 5 for sham operation or *n* = 6 for LCCA ligation group. **(C)** Double *en face* immunostaining against two podosome markers including cortactin (green) and p-SRC-Y416 (red) in the dissected CoW from mice after LCCA ligation for 3 days. **(D)** The percentage of co-localization incidence was measured by the number of yellow-positive puncta over total number of green-positive puncta. More than 120 different cells were examined. *n* = 5 for mice after sham operation or LCCA ligation, respectively. **(E)** The podosome activity was visualized by the released green fluorescence due to the substrate degradation based on *in situ* zymography. See green dots (white arrows) in the endothelium. **(F)** Total fluorescence intensity was quantified by Image J and normalized to that from sham control. More than 12 different frozen sections were examined in either sham-operated (*n* = 6) or LCCA-ligated mice (*n* = 5). White boxed regions were selected for images under higher magnification. Student’s *t*-test used for statistical analysis. ***p < 0.001, **p < 0.01.

It is well established that both MMP14 and MMP2 are downstream executors in podosomes, the organelles located at cell-extracellular matrix (ECM) interaction sites in endothelial cells (Supplementary Figure 8) (Murphy and Courtneidge, 2011). We for the first time revealed the spontaneous assembly of podosomes in brain endothelial cells and confirmed that they are a degradative apparatus and are inducible upon different extracellular cues (Rui et al., 2020). Shear stress was reported to affect podosome formation in cultured umbilical vein endothelial cells (Fey et al., 2016). To examine if HWSS induces podosome formation and function *in vivo*, we developed an *en face* immunostaining method on the CoW. The endothelial cell lining was highlighted by VE-cadherin antibody (red) and podosomes by cortactin antibody (green), a widely used marker for podosomes (Hoshino et al., 2013; Murphy and Courtneidge, 2011). Interestingly, in sham-operated mice, podosomes were barely detected in endothelial cells (Figures 6A1, A3). In contrast, HWSS significantly promoted the assembly of podosomes (Figures 6A2, A4, B). These induced podosomes were further confirmed by co-immunostaining against two markers including cortactin and p-SRC-Y416 (Figures 6C1–C6, D). Although many puncta in endothelial cells were positive for both markers, two subpopulations were noticed that were separately labeled by anti-p-SRC-Y146 and ant-cortactin antibodies. Since SRC is an upstream kinase for cortactin in podosome assembly, the SRC-positive puncta (red signals in Figure 6C3) may represent pre-podosomal sites where cortactin has not been recruited yet.

It was reported that podosome maturation requires the recruitment of MMP14, which in turn activates MMP2 for ECM degradation (Supplementary Figure 8). To evaluate if HWSS-induced podosomes are functional, we performed *in situ* zymography using highly quenched, fluorescein-labeled gelatin based on the published protocol (George and Johnson, 2010; Galis et al., 1995; Mook et al., 2003). Upon proteolytic digestion by MMP2 (gelatinase A), the substrates will release green fluorescence locally. Gelatinase activity is barely detectable in the CoW sections from sham-operated mice (Figure 6E1) but significantly induced in those under HWSS and mainly located in the endothelium (white arrows in Figure 6E2 and quantifications in Figure 6F). All these data support that HWSS induced functional podosome formation and the activation of MMP14–MMP2 axis in podosomes leads to ECM degradation in the CoW, a pathological process frequently manifested in IA lesions.

## Discussion

Through the combined use of HWSS and the strategic administration of a low dose of elastase, we developed a novel mouse model that rapidly forms large IAs with high penetrance. These experimental IAs closely mimic those observed in humans, particularly in terms of outcomes, anatomical locations, and pathological changes. Notably, HWSS-induced early events, such as endothelial podosome formation and extracellular matrix degradation, likely play a key role in HWSS-dependent IA formation in this model, providing new insights into IA pathogenesis.

### A new mouse model of IA that is dependent on HWSS

Over two decades ago, Dr. Nobuo Hashimoto’s team successfully induced IAs in mice by combining unilateral ligation of common carotid artery and renal ligation (Morimoto et al., 2002). To our knowledge, this is the only mouse model that develops IAs in an HWSS-dependent manner. As HWSS is a risk factor for human IAs, the Hashimoto model provides invaluable insight into underlying pathogenic mechanisms. Nonetheless, several limitations associated with this model may impede the discovery of novel molecular mechanisms contributing to the pathogenesis of the disease. First, the sizes of these experimental IAs are small and some of them need to be confirmed by transmission electron microscopy, a technique that may introduce undesired damage to the samples. Second, IAs mainly located at the bifurcation of olfactory artery and anterior cerebral artery. In addition, no data on IA rupture events were mentioned in the report. Therefore, it is likely that a subset of human IAs with a limited spectrum of pathology are mimicked in this model.

Our new model made significant improvements in all the aforementioned areas. Induced IAs are large and can be conveniently identified by the perfusion of blue dye/gelatin mixture, a method to which most labs have access to. In addition, IAs occurred at different or more than one location in the CoW and SAH due to IA rupture was observed. Due to the large size of IAs, we can easily locate the lesion sites and examine their pathology. Following histological and immunofluorescent analyses, we confirmed that these experimental IAs share similar features with those in humans, such as the loss of endothelial integrity, fragmentation of IEL, disorganization of medial layers as well as infiltration of inflammatory cells (Xu et al., 2019; Chalouhi et al., 2012). We also rigorously examined the incidence of IA in each control cohort (single or double surgeries) and demonstrated that our model develops IAs in a HWSS-dependent manner. As such, our model may broadly represent human IAs and thus provide a new paradigm to reveal novel mechanisms that contribute to the disease especially related to HWSS conditions. It is worth noting that HWSS-dependent IA formation has also been observed in other animals, including rats, rabbits, and primates (Uchikawa and Rahmani, 2024). However, these models are either more costly or lack convenient tools for genetic manipulation.

### A robust mouse model that highlights the contribution of endothelial cells to IA

It has been long proposed that endothelial cell dysfunction contributes to IA formation (Sheinberg et al., 2019). Two genetic models supported this hypothesis. Endothelial-specific deletion of Sox17 induces IAs in hypertensive mice (Lee et al., 2015). Our group identified multiple rare variants of THSD1 in IA patients and confirmed that THSD1 suppresses IA formation by regulating endothelial focal adhesion stability (Rui et al., 2017; Santiago-Sim et al., 2016; Kim et al., 2022). Although these genetic models provide invaluable information on the role of endothelial cells in IA pathogenesis, the mechanisms may not be faithfully recapitulated in non-familial IA patients, which constitute over 90% of total IA cases (Vega et al., 2002). It is well established that HWSS is commonly associated with most IA cases (Staarmann et al., 2019). Our model may serve as a useful resource to complement current genetic models, which allows us to identify new pathogenic mechanisms for sporadic IAs especially related to the function and regulation in vascular endothelial cells.

Endothelial cell dysfunction was suggested to be the beginning of IA development (Sheinberg et al., 2019). Indeed, in the first few days after HWSS was introduced, we detected the reduction of basement membrane protein and formation of endothelial podosomes. In contrast, no infiltration of inflammatory cells was found. Consistently, in a HWSS-dependent IA rabbit model, endothelial injury precedes IA formation while inflammatory cells are scant and not localized to the damage sites (Gao et al., 2008). Similarly, endothelial damages of intracranial arteries without inflammation were found at early time points in a HWSS-dependent IA rat model (Kataoka et al., 2020). These results suggest that the priming events that initiate IA development are likely to result from within the arterial wall such as endothelial cell dysfunction rather than inflammation involving the recruitment of leukocytes.

These early events in endothelial cells may be bypassed in the elastase mouse model that also develop large IAs. In this model, a high dose of elastase (35 milliunits) was utilized while local hemodynamic stress was not included (Nuki et al., 2009). Although another modified elastase model included unilateral carotid artery ligation, more than four other manipulations may confound the contribution of HWSS to IAs in their model (Hosaka et al., 2014). Of note, IAs can be found at early time window together with massive inflammation in both models. It is possible that the rapid recruitment of massive inflammatory cells induced by high dose elastase or multifactorial manipulations overrides the contribution of endothelial cells in the early phase of IA. Elastase has also been used in rabbits to induce IAs, producing many pathological features similar to those seen in humans (Wang et al., 2018). This model involves the local incubation of elastase with the common carotid artery without alterations in wall shear stress. Given its reliability in inducing large IAs in the targeted artery segment, it has been widely employed to assess the safety and efficacy of new endovascular devices (Brinjikji et al., 2016). However, it remains uncertain whether shear stress-mediated endothelial dysfunction occurs in this model and contributes to IA development. As such, our new regimen offers a novel approach to exploring the role of endothelial dysfunction in IA pathophysiology. Notably, due to size limitations, our mouse model is not suitable for evaluating the efficacy of endovascular devices, such as stents, as compared to the rabbit elastase model. Nonetheless, considering the cost and accessibility of genetic tools in mice, our model provides an affordable platform for investigating the molecular mechanisms underlying IA pathogenesis.

### Endothelial podosome formation and function as a novel cellular response to HWSS

Podosomes assemble spontaneously in endothelial cells and can be induced by various extracellular cues. Here, we identified HWSS as a novel upstream signal for endothelial podosome formation. Interestingly, in comparison with a basal level of podosome formation in cultured endothelial cells (Fey et al., 2016), endothelial podosomes are barely detected *in vivo*. It is possible that normal shear stress at physiological conditions suppresses podosome formation while deviation such as lack of flow in cell culture stimulates podosome formation. Consistently, impaired shear stress induced podosome formation in human endothelial cells compared to that with a physiological threshold (Fey et al., 2016). These observations together raise an intriguing hypothesis that endothelial podosome formation is a ubiquitous phenomenon in response to abnormal wall shear stress, either low or high, so that vascular wall is remodeled to return the shear stress to its original level. This is in line with the “set point” theory that endothelial cells have a preferred level of shear stress that determines the vascular remodeling (Roux et al., 2020; Baeyens et al., 2016), a process contributing to not only aneurysms but other common diseases like atherosclerosis and hypertension.

The main function of podosomes is to degrade the extracellular matrix. We confirmed that HWSS-induced endothelial podosomes are functional as many degraded spots were noted in the endothelium. Furthermore, the level of fibrillar collagen IV (Triton-insoluble) is significantly reduced. Previous *in vitro* or *ex vivo* studies supported that endothelial podosomes efficiently recognize and degrade collagen IV (Daubon et al., 2016; Rottiers et al., 2009). To our knowledge, our work for the first time provides *in vivo* evidence that endothelial podosomes are shear stress-inducible organelles, which are likely to control basement membrane integrity. Since podosomes are regulated by protein kinases, adaptors and proteases in an orchestrated manner in response to specific stimulus (Hoshino et al., 2013; Murphy and Courtneidge, 2011), this will lead to endothelial ECM degradation in a digest-on-demand pattern. Podosomes are highly regulated, degradative structures that allow the exquisite control of vascular integrity and thus are attractive therapeutic targets. In the future, more experiments are needed to examine if inhibiting podosome formation and function prevents IA formation or improves outcomes. Interestingly, a recent study demonstrated that IAs induced in mice can be treated with sunitinib, a tyrosine kinase inhibitor that blocks PDGFRB function (Shima et al., 2023). Notably, increased metalloprotease levels were detected in these IAs. It is well established that PDGFRB, expressed in vascular mural cells, and PDGF, secreted by endothelial cells, coordinate to maintain barrier function and vascular integrity (Gaengel et al., 2009). Thus, it would be compelling to investigate whether PDGF signaling is dysregulated in endothelial cells in our new mouse model, potentially revealing a novel mechanism underlying podosome formation and function.

Overall, our model highlights the crucial role of endothelial cells in IA development, enabling us to further identify and characterize abnormal flow-regulated signaling pathways that contribute to endothelial dysfunction and disease progression. Emerging techniques, such as single-cell or spatial RNA-seq, hold promise as valuable tools for this investigation. It is important to exercise caution when translating findings from our mouse models to clinical applications, as there is always an inherent gap between animal models and human biology. This limitation is generally applicable to all animal models, including mice. Nonetheless, our mouse model holds significant promise for identifying and characterizing new mechanisms and potential treatment strategies for IA disease.

## Supplementary Material

Supplementary Material

## Data Availability

The original contributions presented in the study are included in the article/Supplementary material, further inquiries can be directed to the corresponding authors.
