# Supplementary material for: High wall shear stress-dependent podosome formation in a novel murine model of intracranial aneurysm
Source: Front Stroke. 2024 Nov 26;3:1494559. doi: 10.3389/fstro.2024.1494559 (PMC11999664; doi:10.3389/fstro.2024.1494559)
Supplement: Supplementary file 1 [file Data_Sheet_1.docx]

**
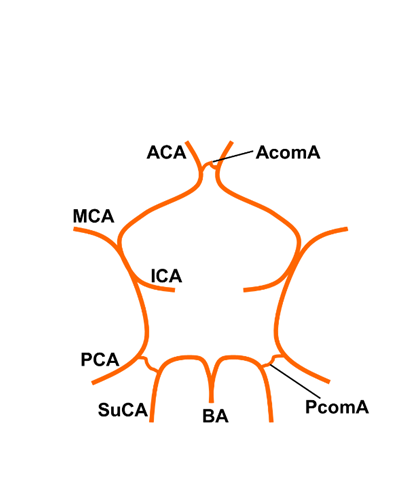
**

**Supplemental Figure 1:** **A schematic model of mouse circle of Willis anatomy**

ICA: internal carotid artery; MCA: middle cerebral artery; PCA: posterior cerebral artery; ACA: anterior cerebral artery; AcomA: anterior communicating artery; PcomA: posterior communicating artery; BA: basilar artery; SuCA: superior cerebellum artery.


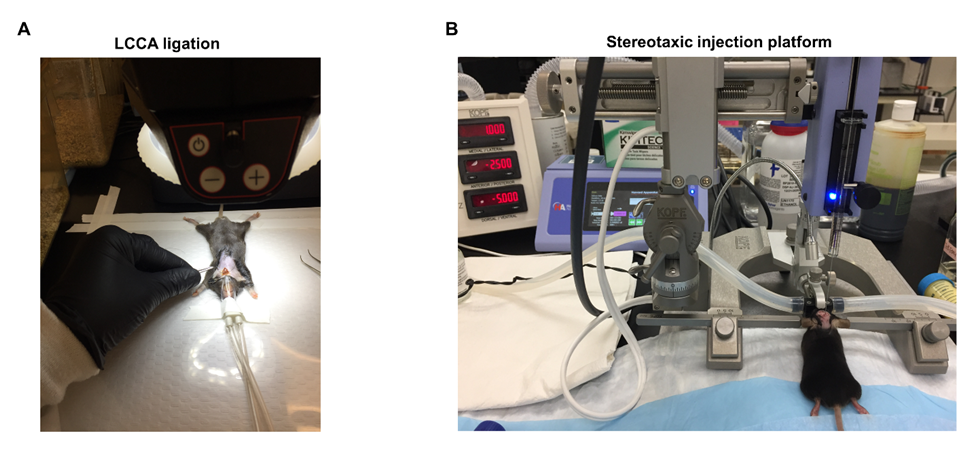


**Supplemental Figure 2: Experimental settings for LCCA ligation and stereotaxic injection in mice.** (**A**) LCCA ligation in mice was performed under Leica S9 stereo microscope. (**B**) An anesthetized mouse was mounted in a rodent stereotaxic frame (David Kopf Instruments). Elastase was injected into mouse basal right cistern using a mini-pump system (Harvard Apparatus).


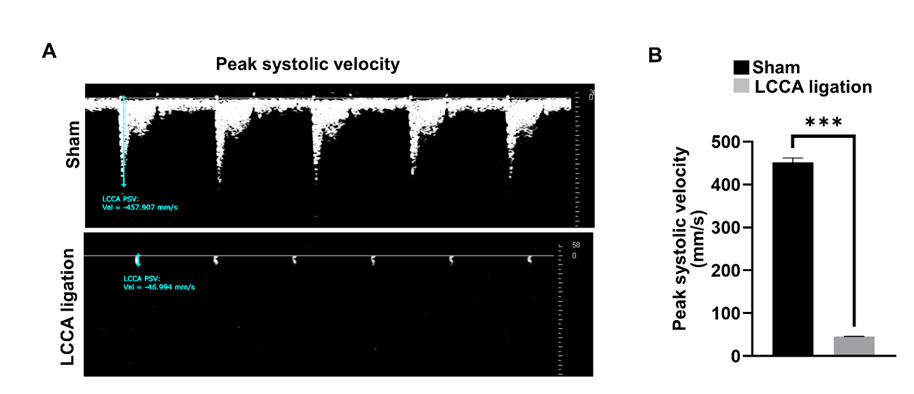


**Supplemental Figure 3: LCCA ligation blocks blood flow in the left carotid artery.**

(**A**) Representative ultrasound images demonstrating peak systolic velocity in the left side of common carotid artery after sham operation or LCCA ligation. (**B**) Statistical analysis of blood flow velocity in LCCA (n=5 for sham-operated mice or LCCA-ligated mice).


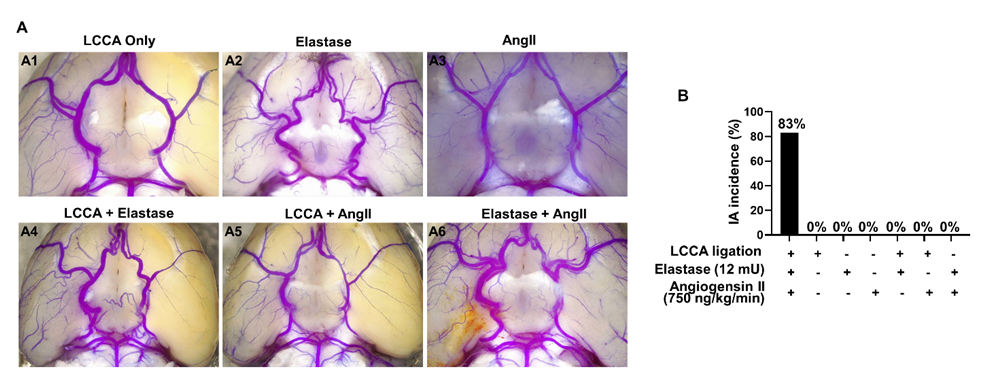


**Supplemental Figure 4:** **Morphological analyses in all control mice**

**(A**) Representative images from the mice that received LCCA ligation (A1), a single dose of elastase (12 mU) injection (A2), infusion of angiotensin-II (750 ng/kg/min) for two weeks (A3), LCCA ligation followed by elastase injection (A4), LCCA ligation followed by angiotensin-II infusion (A5), or the elastase injection followed by angiotensin-II infusion (A6). All mice were perfused with a bromophenol blue dye in 20% gelatin two weeks after the last surgery (n=5 for each group). (**B)** The incidence of IAs in all the above conditions were examined. Note that no IAs were found in any of the control groups.

**
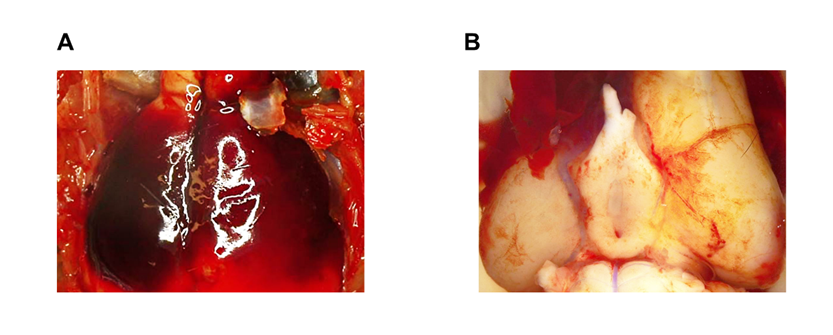
**

**Supplemental Figure 5: IA rupture causes subarachnoid hemorrhage**

(**A-B**) Representative images of subarachnoid hemorrhage were shown in mice after IA induction.


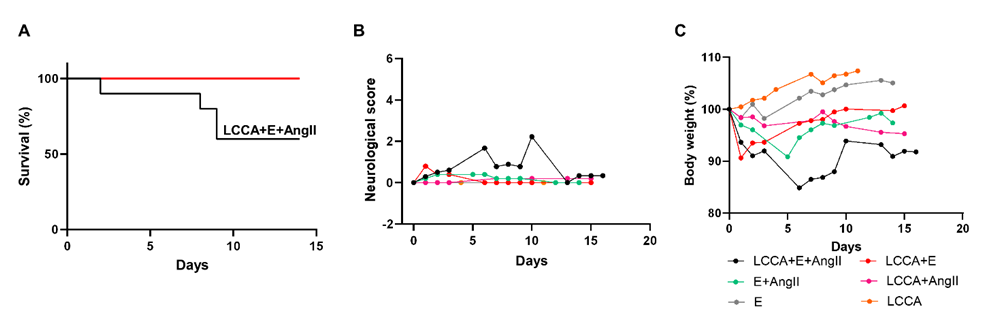


**Supplemental Figure 6:** **IA outcomes in all control mice**

**(A)** No mortality was seen in any of the control groups in comparison with that under our IA induction protocol. (**B-C**) Neurological deficit scores and body weight were recorded daily in all groups. n=10 for LCCA+E+Ang II group and n=8 for each of the rest groups.


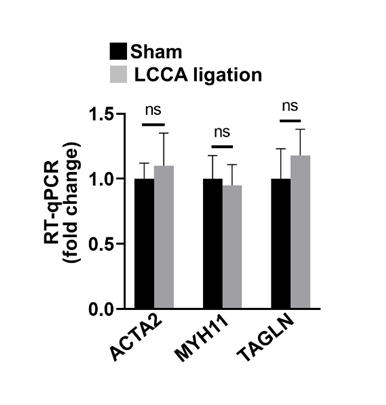


**Supplemental Figure 7: Expression levels of contractile markers for smooth muscle cells**

Quantitative RT-PCR was performed to reveal the expression of ACTA2, MYH11 and TAGLN in the CoW that was exposed to sham operation or LCCA ligation for 3 days. n=3 for each group. n.s. not significant.


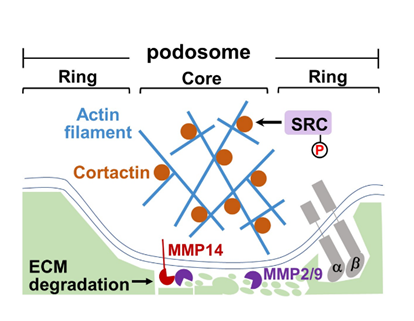


**Supplemental Figure 8: A schematic model of podosome formation and function**

Podosome contains actin enriched core surrounded by a ring structure where integrin adheres to the extracellular matrix. SRC, a non-receptor tyrosine kinase, phosphorylate its substrate cortactin and promotes cortactin-mediated actin assembly in the core of podosomes. MMP14, a transmembrane-type metalloproteinase can be transported to podosomes and in turn activates downstream MMPs such as MMP2 and/or MMP9 for extracellular matrix degradation.
